# Dancing in the dark: no role for consciousness in action control

**DOI:** 10.3389/fpsyg.2013.00380

**Published:** 2013-06-26

**Authors:** Bernhard Hommel

**Affiliations:** Cognitive Psychology Unit, Institute for Psychological Research, Leiden Institute for Brain and Cognition, Leiden UniversityLeiden, Netherlands

## Consciousness and action control

It was Sigmund Freud who put the relative contributions from conscious and unconscious processes to the control of human action on the psychological agenda. Freud (1949) suggested that action control emerges from the interplay between unconscious, automatic, and reward-oriented action tendencies generated by the Id and rational, socially mediated considerations provided by the Ego. While Id processes were assumed to be inaccessible for consciousness in principle, some, but not all Ego operations were claimed to be conscious. This basic logic still provides the blueprint for our current theorizing about action control. Indeed, numerous “dual-route” models in almost all psychological and cognitive-neuroscientific research areas assume that human action emerges from the interplay between consciously inaccessible automatic action tendencies and consciously accessible top-down processes that enforce intentional action goals and social acceptability [for an overview, see Evans and Stanovich (2013)]. Interestingly, many authors associate conscious accessibility with cognitive control (Hommel, 2007). For instance, in the action-control model of Norman and Shallice (1986), automatic, stimulus-driven actions are contrasted with actions that are under “deliberate conscious control,” as if unconscious deliberate control would be inconceivable. In the same spirit, Libet (1985) has suggested that consciousness might have a “veto” that prevents unwanted actions from execution. In the following, I will argue that there is no evidence that consciousness plays a causal or decisive role in action control, so that there is no reason to believe that consciousness is necessary or useful for the control of human actions.

## Executive ignorance

If agents would control their actions through the having of conscious experiences, they should be able to report how and based on what information they are exerting such control. However, agents know surprisingly little about their actions (Wegner, 2002). As James (1890, p. 499) put it: “we are only conversant with the outward results of our volition, and not with the hidden inner machinery of nerves and muscles which are what it primarily sets it at work”—a kind of executive ignorance (Turvey, 1977). Numerous examples show that actions can be parameterized and redirected by stimuli that the agent is unable to consciously perceive because of subliminal presentation or lesions in higher perceptual areas [e.g., Prablanc and Pélisson, 1990; for overviews, see Glover (2004); Milner and Goodale, 1995]. Agents can be easily fooled into experiencing artificial effectors as being a part of their own body (Botvinick and Cohen, 1998) and perceiving actions of other people as being carried out by themselves, or vice versa (e.g., Nielsen, 1963). But even higher-level executive-control operations can be triggered by stimuli the conscious perception of which is prevented by masking procedures [for overviews, see van Gaal et al. (2012); Kunde et al., 2012]. Among other things, this holds for the implementation of a task set (Reuss et al., 2011) and the stopping of the planned action (van Gaal et al., 2010), suggesting that executive control does not rely on consciousness. Indeed, there is widespread agreement that generating a conscious experience takes time, at least 300-500 milliseconds (e.g., Libet, 2004; Dehaene et al., 2006), which would be way too slow for many everyday actions and most reactions in cognitive-psychological tasks. Hence, not only is our conscious knowledge about action control severely limited, it is also difficult to see at which point in time the application of this knowledge would be useful.

One way to save a role for consciousness and action control would be to relate it to the translation of intentions into more specific action plans. Consider, for instance, the seminal study of Libet et al. (1982), who observed that the physiological indicators of action preparation preceded the agent's conscious urge to act by hundreds of milliseconds. Even though this might be taken against a causal role of conscious experience in the online-generation of actions (Wegner, 2002), it does not rule out such a role in translating instructions into a general action plan at the beginning of the study. Indeed, several authors since Exner (1879) have considered that implementing such a plan delegates control to internal and external stimuli, which might very well operate outside of consciousness (Bargh, 1989; Hommel, 2000). And yet, this would leave a severely limited role of consciousness that is restricted to off-line control. Moreover, it has been claimed that integrating information from and across different informational maps and systems require conscious experience (Baars, 1988) and one might argue that preparatory off-line action planning involves such kind of cross-domain integration. However, recent demonstrations that neither the integration of multimodal event features (Zmigrod and Hommel, 2011) nor the integration of objects with their background (Mudrik et al., 2011) depend on consciousness do not support this possibility.

## Lack of specificity

While many action-control operations were shown to be independent of conscious experience, some have been claimed to require consciousness. After reviewing the available evidence van Gaal et al. (2012) conclude that the conscious representation of task-related stimuli increases the duration (in the range of milliseconds or seconds), flexibility, and strategic use of the represented information for action control and other cognitive operations. In another recent review, Kunde et al. (2012) conclude that all sorts of cognitive-control operations can be automatically driven by endogenous information as long as it is provided by individual, clearly discriminable events and associated with the operation in a one-to-one manner. Conscious representation, in turn, is required if the cues are implicit, distributed in space and time (like frequency information), and context-dependent. Key findings emphasized in both reviews is the absence of conflict-induced cognitive adaptations, like post-error slowing and increased attention to relevant information after conflict trials or frequent conflicts, if the conflict is not consciously perceived.

It is interesting to note that these examples are not only surprisingly few (if one would suspect consciousness to control action as a rule) but they are also rather nonspecific and nonrepresentative for voluntary-action control. They are not representative because sessions with tens to hundreds of trials with many repetitions of just a few stimuli and responses are necessary to create these (often rather small) trial-to-trial effects, conditions that under real-life conditions would motivate the employment of automatic routines rather than online action planning (Norman and Shallice, 1986). And they are nonspecific as any kind of consciously represented information—not just action-related one—is more likely to be held active and made available for a longer time. Most importantly, none of the consciousness-related abilities considered so far (information maintenance, availability, and conflict-induced adaptations) seems so crucial that its loss would seriously compromise voluntary action control.

## No causality

Cognitive operations or processing results may correlate with the presence or absence of conscious representation for many reasons. The probably most obvious one has to do with the fact that human brains are noisy, so that the quality of representing a particular piece of information can vary over time and trials. Signal-detection theory states that reporting a particular state of affairs requires that evidence passes a particular threshold and that it can be distinguished from noise, which implies that low-energy, complex, and/or difficult-to-discriminate information is unlikely to be reportable. But it also implies that this information is unlikely to be usable for other purposes than conscious report, irrespective of any causal dependency of that other purpose on conscious experience. Accordingly, the mere correlation between the accuracy of conscious report and the usability of information for action control does not tell us anything about the causal relationship between consciousness and action control.

What is needed to make a causal case is the demonstration that preventing conscious representation without reducing signal quality or affecting thresholds impairs action control, or some other sort of proof that signal quality and threshold-setting cannot account for the correlation. According to my knowledge, no such proof has been provided so far. Worse, there is not even evidence that the few consciousness-correlated functions are really under voluntary control. Observations like post-error slowing or increased attention to relevant information after incongruent trials are often called “strategic” because they seem to optimize some aspect of behavior: slowing down after having done something wrong and paying attention after having experienced decision conflict sounds very reasonable and makes the impression of being the outcome of a strategic (i.e., goal- and context-dependent) decision. However, not only are the overall performance benefits of such “strategies” often negligible (speed is traded for accuracy with post-error slowing and facilitation benefits are traded for interference costs through post-conflict potential effects), but agents also seem to have little choice in applying them. As shown by Jiménez and Méndez (2013), the impact of previous incongruency experiences is entirely independent of (i.e., unaffected by) the actual expectations of the agent, suggesting that sequential effects are due to an automatic learning process [for an application of this logic to congruence-probability effects, see Hommel (1994)]. The degree of associative learning and the reliability of the emerging associations must depend on the quality and discriminability of the signals being associated, which would explain why sequential effects are less pronounced and less reliable under conditions that are likely to reduce signal-to-noise ratios and discriminability—Kunde et al.'s (2012) “implicit” stimulus conditions. Hence, the available evidence can be parsimoniously accounted for by well-understood low-level associative processes. These processes apparently run off automatically and, even though they might often support action control (which might well be the reason why evolution has equipped us with them), they can hardly count as “strategic” except in a metaphorical sense (much like Darwinian evolution would be considered a survival “strategy”). Most importantly, there is not any positive evidence that they can be “consciously controlled” and the little evidence we have actually suggests the opposite.

## What else is consciousness good for?

Taken altogether, there is not yet any demonstration of a causal role of consciousness in human action control, which given the enormous interest in this issue must be considered surprising. And it raises the question what else conscious experience might be good for. Even though this brief opinion paper does not seem appropriate to even try tackling that issue exhaustively, a few speculations might be in order. The most obvious difference between conscious and unconscious representations is that we are commonly able to communicate about the former but not the latter. Indeed, most researchers accept communicability of the represented information as either the consciousness-defining characteristic or at least a useful experimental operationalization. It is hard to see what communicability might contribute to the online control of actions but it provides obvious benefits for social purposes: we can inform other people about our action plans, instruct others to carry out particular actions, evaluate these actions and provide feedback, and discuss the pros and cons of alternative action plans. All of that effectively increases social predictability and thus reduces uncertainty—an aversive state that is driving much of our behavior (Berlyne, 1960). Communicability might also help us to describe and try understanding our own behavior in ways that allows relating and comparing it to others, thus providing the opportunity for self-reflection and social impression management. Most of these hypothetical functions are post-actional, so that they are not compromised by the long time that conscious representation needs to build up or by the lack of impact of most if not all conscious representations on action control proper. And they are not unlikely to work back on action control in a broader, socially embedded sense: how we interpret and sell our actions to the public will affect its reactions and feedback, which again might often provide selective reward and social constraints for our future actions. Hence, the true impact of consciousness on the control of our actions may be more indirect and more socially mediated than common sense has it.
